# Bibliometric analyses of turnover intention among nurses: implication for research and practice in China

**DOI:** 10.3389/fpsyg.2023.1042133

**Published:** 2023-06-15

**Authors:** Huifang Zhang, Li Ping Wong, V. C. W. Hoe

**Affiliations:** Centre for Epidemiology and Evidence-Based Practice, Department of Social and Preventive Medicine, Faculty of Medicine, University of Malaya, Kuala Lumpur, Malaysia

**Keywords:** bibliometric, nurses, turnover intention, status, trend

## Abstract

**Objective:**

The aim of this study was to analyze the current status of research on nurses’ turnover intention and to provide suggestions and references for promoting research on turnover intention and for promoting hospital talent development.

**Methods:**

We used the bibliometric method “turnover intention” or “intention to leave” and “nurse*” as subject terms, and 1543 articles from 2017 to 2021 were retrieved from the WoS database using VOSViewer and CiteSpace software. Article based on this descriptive statistical analysis was performed on the year of publication, region, institution, journal of publication, and cited articles.

**Results:**

A total of 1,500 articles met the inclusion criteria. There is an overall upward trend in the number of articles published in the field of nursing in terms of turnover intention from 2017 to 2021. The United States has the highest number of publications and the highest number of institutions, while China ranks second in terms of publications, but there are no Chinese research institutions in the top 10. The top three journals in terms of the number of articles published are the Journal of nursing management, the Journal of advanced nursing, and the Journal of clinical nursing; Oman’s League had the highest number of citations for their article in 2021; the most frequently occurring keywords are burnout, stress, satisfaction, model, work environment, organizational commitment, perception, predictor, mental health, and mediating role.

**Conclusion:**

There is a great need for further research on how to develop sound measures to tackle nurse turnover intention. The following improvements should be made, such as to enhance research institutional settings for nurses’ turnover intention in China and to increase attention to nurse burnout and possible mediating influences in future studies.

## 1. Introduction

In recent years, the shortage of nurses due to turnover has become a global problem (Hayes et al., 2006). Their departure exacerbates the serious shortage of nursing human resources, which affects the quality of care and patient safety and the development of nursing careers (Tuckett et al., 2015; Gray et al., 2018; Ryan et al., 2019). With the rapid growth of the world population and the strengthening of the aging trend, people’s living standards are improving, and the requirements for healthcare are getting higher, especially for the quality of nursing services (Qiqi et al., 2021). Therefore, how to reduce the nurse turnover rate and stabilize their workforce has become an urgent problem for their managers to solve. Willingness to leave is the best predictor of turnover (Hom et al., 1979). In terms of the use of research tools, the current research articles on nurses’ turnover intention are vast, extensive, and mixed. Using traditional literature search methods to collect and organize literature related to this topic through the general academic search tool, it is easy to miss important literature on the topic. A comprehensive and systematic analysis in the form of mapping is conducted with the help of the CiteSpace visual metric analysis tool, which improves the research efficiency of researchers by providing macroscopic perceptions and predictions of research content from a quantitative perspective. In addition, there is a gap in the current research on bibliometric analysis conducted on nurses’ turnover intention. Therefore, this study uses bibliometric analysis to sort out and analyze global research results related to nurses’ turnover intentions from 2017 to 2021, to provide a certain database, to fill the gap of turnover intention research analysis based on bibliometric methods, to provide a comprehensive grasp of the progress of current research, and to provide a reference for predicting future research related to nurses’ turnover intention.

## 2. Objects and methods

### 2.1. Objects

#### 2.1.1. Inclusion criteria

Our study was based on the Web of Science (WoS) database, which is one of the most widespread databases in different scientific fields which are frequently used for searching the literature (Joshi, 2016). Various scholars for bibliometric evaluation across the sciences, social sciences, and arts and humanities to find the high-quality research most relevant to our area of interest.

#### 2.1.2. Exclusion criteria

The following studies were excluded: (a) duplicate publications and (b) those whose content was not relevant or of little relevance to the topic of nurses’ turnover intention.

#### 2.1.3. Searching strategy

The retrieve strategies were as follows: TS = (“turnover intention “or “intention to leave “and “nurse*”). The time span is from January 2017 to January 2022. The implementation search was conducted from 10 September 2022 to 11 September 2022. The type of literature included “Article,” “Review,” and “Editorial.” Language = English.

#1 turnover intention: (“turnover intention”)OR (“intention to leave”).

#2 Nurse:(Nurse*).

#3 Combination:#1AND#2.

#### 2.1.4. Study selection

The database search was performed on 2 days to avoid the possibility of introducing bias due to the daily citation updates. Two members of our research team (zhf和wjx) independently assessed the retrieved documents, including journals, institutions, and countries. If there are differences of opinion, they are resolved by consensus.

#### 2.1.5. Searching results

A total of 1,500 articles met the inclusion criteria. According to the retrieve strategies, a detailed search for publications obtained 1,543 articles from the Web of Science (“WoS”), in which one duplication was deleted, leaving 1,542 articles. Afterward, 42 articles were removed due to their unmatched key terms after careful screening.

#### 2.1.6. Data abstraction

The following data were retrieved from the included articles, including publication time, institutional information, regional information, journal publications, and cited articles.

### 2.2. Methods

#### 2.2.1. Bibliometric analysis

To save the information of the search results, we used the search analysis function provided by the WoS database and compared the title, author, abstract, and content to exclude duplicates, invalid, or incomplete papers. In addition, keywords with the same prefix but not focused on the intention of leaving the nursing field were excluded. Analysis of the most cited articles by journal, region, institution, and the number of citations predicted important trends in research. When analyzing the data, the words or phrases with the same or similar meanings in the included literature were combined into one keyword. For example, “turnover intention” stands for “intention to leave,” and “nurse” stands for “nursing,” “nurses,” etc. Journal impact factors are based on the latest data from the Web of Knowledge JCR (2021). The keywords with higher frequency reflect the hot spots of research in the field, so the keywords are regarded as the concentration of research hot spots in the field. These high-frequency words are analyzed by network clustering, and the clustering effect is presented in the form of a graph; the higher the frequency, the more closely related the group of words. Bibliometric mapping were performed using the VOSViewer software (Leiden University, the Netherlands). VOSViewer uses text mining to recognize publication terms and then employs the mapping technique called visualization of similarities (VoS), which is based on co-word analysis, to create bibliometric maps or landscapes (Kokol et al., 2022).

#### 2.2.2. Cluster analysis

CiteSpace software is a tool that identifies and displays new trends and developments in science in the scientific literature, which is used to find research advances and current research frontiers in a subject area and their corresponding knowledge base. The main functions include research subject and region analysis; mediated centrality is a measure of the importance of nodes in the network, and the higher the value, the greater the importance. In this study, the keywords are counted, and the topics are clustered and analyzed.

## 3. Results

### 3.1. Original articles reached

**Figure 1:**
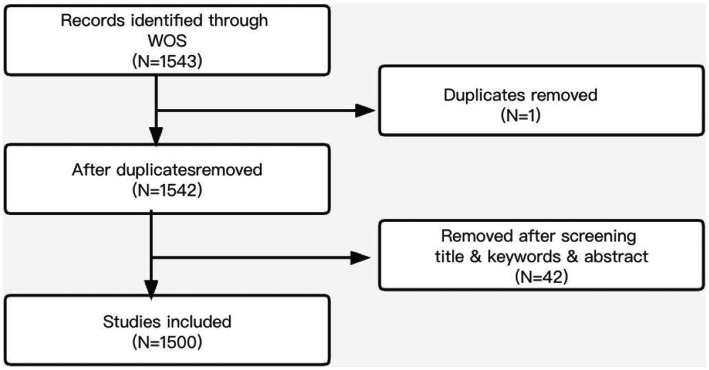
PRISMA flow chart.

**Figure 2:**
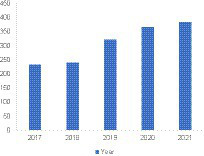
Number of postings on nurses’ turnover intention from 2017 to 2021.

An analysis of trends in the volume of literature provides a perspective on the level of attention and trends in research related to nurses’ turnover intention (Zhou et al., 2011). The preferred reporting items for systematic reviews and meta-analysis (PRISMA) diagram explains how articles are selected (Mohera et al., 2009). In this study, PRISMA guidelines guided the process of systematic review (Figure 1). They are respectively 233,240,321,365 and 384 from 2017 to 2021, There ia a sharp increase after 2019 (Figure 2), in which 1 duplication were deleted, leaving 1542 articles. Afterward, 42 articles were removed due to their unmatched key terms after careful screening. Therefore, a total of 1500 articles were included and saved for the next step in the process.

### 3.2. Order of productive affiliates

The distribution of institutions may be related to the and high priority given to nursing staff turnover in these regions. The contributions of the 10 most productive institutions are ranked (Table 1). University of California System, in which the publication number ranked first (32), followed by University of Texas System. Sultan Qaboos University (“SQU”) published the highest percentage of cited articles. Among them, seven were from university institutions, eight were from the United States, one was from Sudan, and one was from Canada.

**Table 1:** Top 10 highly prolific research institutions from 2017 to 2021.

Rank	Institution	Number	Times cites	Percent
1st	University of California System, US	32	494	2.84%
2nd	University of Texas System, US	28	419	2.41%
3rd	Harvard University, US	28	314	1.80%
4th	US Department of Veterans Affairs, US	27	451	2.59%
5th	Veterans Health Administration VHA, US	26	366	2.10%
6th	University of North Carolina, US	23	252	1.45%
7th	Sultan Qaboos University, Sultanate of Oman	21	646	3.71%
8th	University of Wisconsin System, US	20	261	1.50%
9th	University of Toronto, Canada	19	238	1.37%
10th	Pennsylvania Commonwealth System of Higher Education (PCSHE), US	18	231	1.33%

### 3.3. Order of productive regions

An analysis of the geographical distribution provides insight into the direction and development of research on nursing turnover intention in various countries around the world (David Mohera et al., 2009). The retrieved articles were from 92 countries, of which the United States ranked first, followed by China, Australia, and Canada (Table 2). The number of published articles in the United States exceeds that of China by about three times, and the academic research capacity maintains a leading position, with 10 for developed countries and six for developing countries.

**Table 2:** Top 10 productive corresponding author countries from 2017 to 2021.

Rank	Country	Number	Percent
1st	US	532	35.47%
2nd	China	154	10.27%
3rd	Australia	124	8.27%
4th	Canada	115	7.67%
5th	South Korea	88	5.87%
6th	UK	84	5.60%
7th	Iran	49	3.27%
8th	Sweden	43	2.87%
9th	Taiwan (China)	42	2.80%
10th	Saudi Arabia	41	2.73%

### 3.4. Order of productive journals

The quality of the literature can be reflected, to some extent, by the journals in which the literature is published. Articles of inclusion were published in 306 different journals, of which the Journal of Nursing Management ranked first (112,7.47%), followed by the Journal of Advanced Nursing (63,4.20%) and the Journal of Clinical Nursing (493.27%) (Table 3). The International Journal of Nursing Studies established by Elsevier, United Kingdom, is an internationally renowned SSCI and SCI double-search journal, (Li Yule and Xinjuan, 2009) which is ranked in the JCR SSCI division in the Nursing category, Region 1. Most of these journals focus on research related to nursing medicine.

**Table 3:** Top 10 productive journals turnover research from 2017 to 2021.

Rank	Journal names	Number	Percent	IF
1st	Journal of Nursing Management	112	7.47%	4.68
2nd	Journal of Advanced Nursing	63	4.20%	3.057
3rd	Journal of Clinical Nursing	49	3.27%	4.423
4th	International Journal of Environmental Research and Public Health	44	2.93%	4.614
5th	Journal of Nursing Administration	34	2.27%	4.423
6th	BMC Health Services Research	28	1.87%	2.908
7th	International Journal of Nursing Studies	27	1.80%	6.612
8th	BMJ Open	23	1.53%	3.006
9th	Nursing Open	23	1.53%	1.942
10th	BMC Nursing	22	1.47%	3.189

### 3.5. Order of active cited articles

Citation frequency is an important indicator used in bibliometrics to measure the social salience and academic impact of academic papers (Yang and Cai, 2010). The 10 most cited articles in the field of turnover intention are shown in Table 4. The most frequently cited article was “Fear of COVID-19, psychological distress, work satisfaction and turnover intention among frontline nurses” by Labrague, published in 2021 in the Journal of Nursing Management. This article has been cited more than 150 times, followed by “The role of job satisfaction, work engagement, self-efficacy, and agentic capacities on nurses' turnover intention and patient satisfaction” by De Simone, S et al., published in 2018 in Applied Nursing Research. Both are cited more than 2000 times. All of these top articles were published in high-impact journals and shared an average citation number of 1,265. In total, 90% of the articles were published in 2020. Among them, eight are JRC Q1, all of them are high-impact journals in this field, and two are from China.

**Table 4:** Top 10 cited articles on turnover intention research from 2017 to 2021.

Rank	Article title	Journal	Times cites	Year	IF/JCR
1	Fear of COVID-19, psychological distress, work satisfaction and turnover intention among frontline nurses, Oman	Journal of Nursing Management	199	2021	4.68 (Q1)
2	The role of job satisfaction, work engagement, self-efficacy and agentic capacities on nurses' turnover intention and patient satisfaction, Italy	Applied Nursing Research	143	2018	1.847 (Q1)
3	Effects of nurse work environment on job dissatisfaction, burnout, intention to leave, Thailand	International Nursing Review	132	2017	3.384 (Q1)
4	Workplace violence, job satisfaction, burnout, perceived organizational support and their effects on turnover intention among Chinese nurses in tertiary hospitals: a cross-sectional study, China	BMJ Open	94	2018	3.006 (Q1)
5	Relationships between burnout, turnover intention, job satisfaction, job demands and job resources for mental health personnel in an Australian mental health service, Australia	BMC Health Services Research	93	2019	2.908 (Q2)
6	Factors predicting Registered Nurses’ intentions to leave their organization and profession: A job demands-resources framework, New Zealand	Journal of Advanced Nursing	91	2018	3.057 (Q1)
7	Moral distress in physicians and nurses: impact on professional quality of life and turnover, USA	Psychological Trauma-Theory Research Practice and Policy	90	2017	9.398 (Q1)
8	The determinants and consequences of adult nursing staff turnover: a systematic review of systematic reviews, UK	BMC Health Services Research	86	2017	2.908 (Q2)
9	The relationship between job satisfaction, work stress, work–family conflict, and turnover intention among physicians in Guangdong, China: a cross-sectional study, China	BMJ Open	86	2017	3.006 (Q1)
10	Relationship between ethical work climate and nurses’ perception of organizational support, commitment, job satisfaction and turnover intent, Egypt	Nursing Ethics	85	2017	3.344 (Q1)

### 3.6. Keywords and cluster analysis

**Figure 3:**
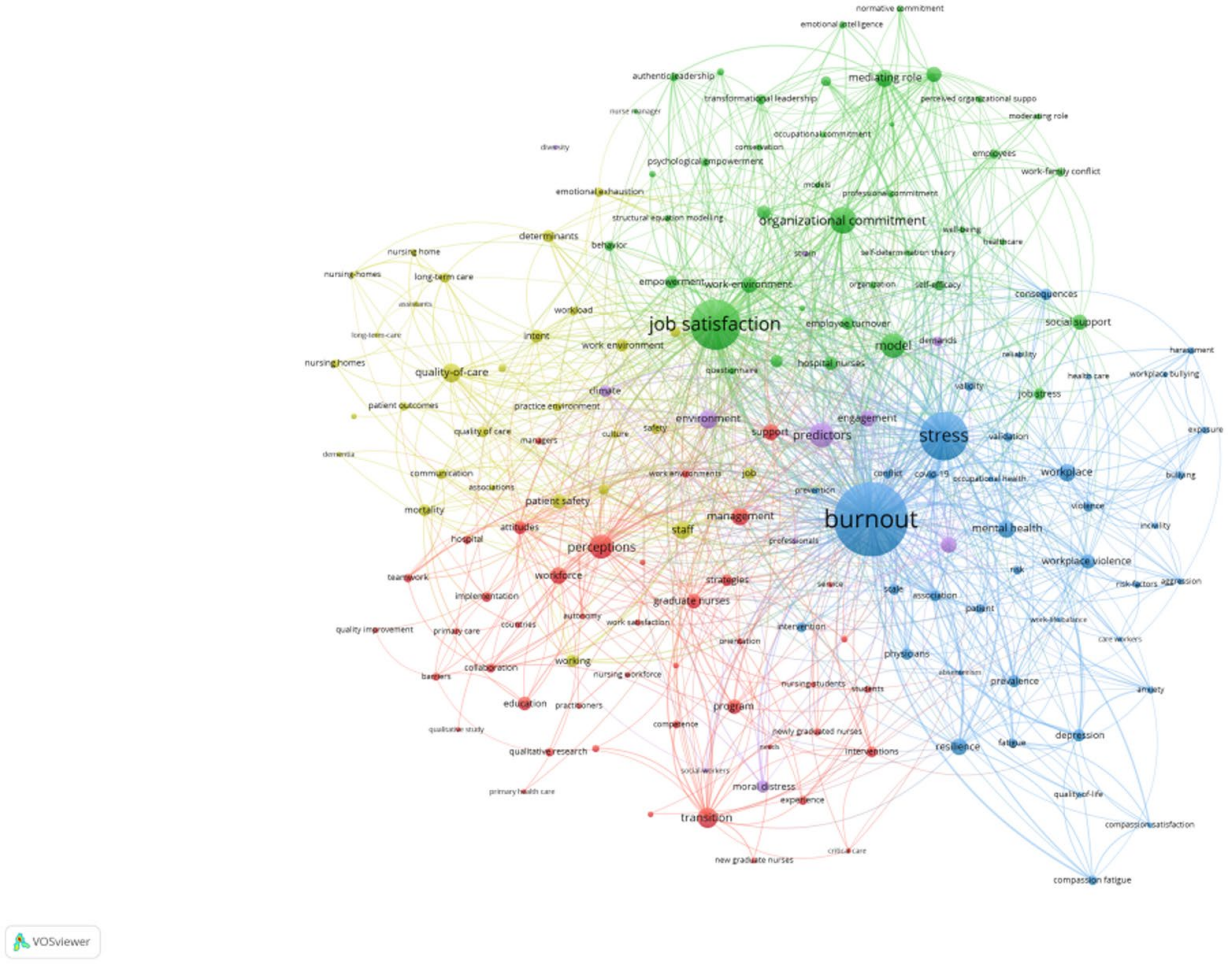
Hotspot distribution in the field of nursing from 2017 to 2021.

The keywords are a general summary of the article content, from which the overall research content and research concerns can be seen. In this study, we extracted and analyzed 9,141 keywords, in addition to the subject keyword of turnover intention and nurses, some of the keywords are closely related synonyms, and the top 10 keywords with higher frequency are in order of appearance: burnout, stress, job satisfaction, model, work environment, organizational commitment, perception, predictor, mental health, and mediating role (Table 5). It is shown that nurse burnout is still a hot research topic (Figure 3).

**Table 5:** Top 10 keywords in the field of nursing from 2017 to 2021.

Rank	Keywords	Number	Centrality	First annual
1	Burnout	254	0.02	2017
2	Stress	181	0.01	2017
3	Job satisfaction	155	0.00	2017
4	Model	97	0.01	2017
5	Work environment	97	0.01	2017
6	Organizational commitment	95	0.01	2017
7	Perception	86	0.01	2017
8	Predictor	83	0.01	2017
9	Mental health	55	0.01	2017
10	Mediating role	55	0.02	2017

According to the domain to which each cluster belongs, the keyword cluster analysis is divided into four clusters, the general directions of which can be divided into job satisfaction, mental health, strategy, and teamwork (Table 6).

**Table 6:** Keyword clustering groups in the field of nursing from 2017 to 2021.

Clusters	Number	Name	LLR
A	#0	Job satisfaction	Commitment, performance, workplace, environment, and justice
B	#1	Mental health	Stress, burnout, depression, disease, and quality of care
C	#2	Strategy	Model, factors, mediating role, predictor, and management
D	#3	Teamwork	Leadership, staff, employee, perception, and empowerment

## 4. Discussion

The data show a positive trend of increasing articles published on turnover intentions during the last 5 years, although there is a slow rise in 2017–2018, but still slowly increasing articles, and an active period in 2019–2021, with a surge in articles and increased attention, which is consistent with Naila Ufiidatul Uzkiyyah study (Uzkiyyah et al., 2022), suggesting nursing managers and researchers became to pay attention to the problem of nurses’ turnover intention. The reasons might be associated with the shortage of nursing human resources, and the orientation of national policy (Li and Liu, 2011; Li et al., 2015). Turnover intention among nursing staff is a growing and highly visible issue globally (Li and Liu, 2011; Li et al., 2015; Lyua et al., 2016). In addition, due to the long-term pandemic that has taken over almost the entire world, the intention to leave is starting to occur in various countries and is expected to increase by the end of 2023.

Institutions can be classified as government departments, research institutions, universities and affiliated hospitals, the Centers for Disease Control and Prevention (CDC), and others. Among the top 10 institutions, universities account for 70% of research output, indicating that universities are the most dynamic and creative. Among them, there is consistency with NU studies showing that research results mostly occur in universities. As an important educational and research institution, the university assumes an important role in the training of nurses and is more able to closely combine practical and theoretical aspects and use the practice to improve the quality of nurse training. However, the most productive institutions were the University of California System in the United States, a consortium of 10 universities located in California, United States. It is the largest university system in the state and one of the most influential public university systems in the world. It is also possible and closely related to the mass departure of nurses in the United States, which has been reported in recent years (Buerhaus et al., 2022). The highest percentage of cited frequency of articles published by SQU may be related to its first cited frequency of articles published in 2021. This is a reflection of the efforts and high impact of foreign research on burnout. The United States and China were the most productive countries, contributing more than 686 (45.74%) articles. This is also consistent with Naila Ufiidatul Uzkiyyah’s ranking of the top two countries contributing the most to research results. Australia and Canada also topped the list, ranking third and fourth, respectively. The United States has been critical in promoting and participating in turnover intention research, early start, and is related to the intensity of its economic impact, and scientific and technological strength. In addition, China is one of the country most severely affected by nursing departures and has a late start in research, requiring great efforts to stabilize the nursing workforce, but the fact that many articles are published in Chinese and that China promotes writing in its own country has likely led to a significant lag in the number of articles published in the United States. They have played a very important role in the research and academic exchange of nurses’ turnover intention.

The journals that published the articles were mainly from the more influential journals in the field of nursing. The journal impact factor is an important indicator of the influence of academic journals, and the larger the impact factor, the higher the average citation rate of journal papers, and the higher the international recognition. As a carrier of the research progress of the intention to load separation, it promotes the solution of the talent pool stability program. This is consistent with the Su Yanbing study, where nursing human resource management has been a popular topic in the Journal of Nursing Management (Yanbing et al., 2019). This indicates that research on nurses’ turnover intention has received attention from high-level journals. Therefore, researchers can track and pay attention to the abovementioned key journals to keep abreast of the latest developments in the current research on turnover intention, predict the direction of research development, and increase at the same time when publishing relevant research results, they can choose journals that are consistent with the research content.

The top ranking 2021 of highly cited papers came from Oman, and the articles were published during COVID-19, indicating that the outbreak was followed by a rapid response from Oman researchers and that nurse turnover intentions were given high priority in that country. Two of these articles were from China, demonstrating that the issue of turnover intention remains a hot topic of research in China, but that there has been a relative lack of empirical research in recent years. We found that studies related to nurses’ intention to leave covered topics related to job satisfaction and mental health and that burnout and regulatory roles were major concerns in the turnover intention studies. Burnout among nurses remains a hot topic. This coincides with Huiyun Yang, where nurses’ intention to leave is influenced by a variety of factors such as burnout and job stress (Yang et al., 2017). Burnout includes three major components: emotional exhaustion, depersonalization, and low personal fulfillment (Galdino et al., 2021). It usually occurs in the final stage of work stress and can be said to be a long-term, multifaceted result of work stress, leading to a psychological state of fatigue and apathy, which is a key factor forcing nurses to leave the profession. Burnout is a complex issue and an important predictor of the modern workplace, and its prevalence has increased dramatically in recent years, especially among nursing professionals (Leiter and Maslach, 2009; Cañadas-De la Fuente et al., 2015). Some scholars intervened to improve nurses’ time allocation by implementing time management methods and improved nursing scheduling patterns to reduce nurses’ burnout and thereby alleviate turnover intentions (Şenormancı et al., 2014). Therefore, to make nursing work more orderly and reasonable and reduce nursing workforce attrition, it is necessary to pay attention to nursing staff burnout and give timely help.

Research on the mediating factors of turnover intention may be the future research direction (Shah et al., 2021). There is a relative lack of empirical research on the mediating factors of turnover intention, interventions in terms of external organizational teams and internal self-perceptions, and developing a favorable working atmosphere, creating a healthy public opinion environment, strengthening nursing staff’s learning, and promoting social support in healthcare institutions (Yang et al., 2022). It suggests that nursing researchers should expand the depth and breadth of research on nursing turnover intention.

## 5. Conclusion

Bibliometrics is a multidisciplinary interdisciplinary discipline based on bibliography, information science, mathematics, and statistics (Poghosyan et al., 2009; Thompson and Walker, 2015; Xuang et al., 2021). CiteSpace software is a tool to visualize literature information data (Peter et al., 2021), which can not only assist in analyzing research hotspots in the nursing field about the intention to leave for clustering analysis but also predict the trend of theme evolution and prepare nurses in advance. Our study made a comprehensive analysis of all included articles and showed the present status, focus, and perspectives of research on nurse turnover intention between 2017 and 2021 and provided a reference for future related in-depth research. Currently, there is still a gap between the setting of influential research institutions engaged in the field of turnover intention in nursing in China and the international level, job burnout in nursing may still be a research hotspot for some time, and the focus of turnover intention research on the mediating factors of nursing staff may be the focus of future attention.

## 6. Limitations

One of the limitations is that the analysis was limited to publications indexed in WoS which does not index all journals, so articles from other databases (e.g., Scopus and PubMed) may have been overlooked. In addition, we only included publications in English, which may introduce publication bias. For example, the official languages are Chinese and Arabic in China and Saudi Arabia, and many cases are published in local language.

## Data availability statement

The original contributions presented in the study are included in the article/supplementary material, further inquiries can be directed to the corresponding authors.

## Author contributions

HZ responsible for the writing, conception and design of the article, and data collection. LW and VH provided critical review of the intellectual content of the article and corresponding work support. All authors contributed to the article and approved the submitted version.

## Conflict of interest

The authors declare that the research was conducted in the absence of any commercial or financial relationships that could be construed as a potential conflict of interest.

## Publisher’s note

All claims expressed in this article are solely those of the authors and do not necessarily represent those of their affiliated organizations, or those of the publisher, the editors and the reviewers. Any product that may be evaluated in this article, or claim that may be made by its manufacturer, is not guaranteed or endorsed by the publisher.
